# Effect of Time Since Death on Multipathogen Molecular Test Results of Postmortem Specimens Collected Using Minimally Invasive Tissue Sampling Techniques

**DOI:** 10.1093/cid/ciab810

**Published:** 2021-12-15

**Authors:** Jeanette Dawa, Edwin Walong, Clayton Onyango, John Mathaiya, Peter Muturi, Milka Bunei, Washington Ochieng, Walter Barake, Josilene N Seixas, Lillian Mayieka, Melvin Ochieng, Victor Omballa, Shirley Lidechi, Elizabeth Hunsperger, Nancy A Otieno, Jana M Ritter, Marc-Alain Widdowson, Maureen H Diaz, Jonas M Winchell, Roosecelis B Martines, Sherif R Zaki, Sandra S Chaves

**Affiliations:** 1 Washington State University, Global Health Programs (Kenya Office), Nairobi, Kenya; 2 College of Health Sciences, University of Nairobi, Nairobi, Kenya; 3 Division of Global Health Protection, Centers for Disease Control and Prevention, Nairobi, Kenya; 4 Department of Pathology, Thika Level 5 Hospital, Kiambu County, Kenya; 5 Infectious Diseases Pathology Branch, Division of High-Consequence Pathogens and Pathology, National Center for Emerging and Zoonotic Infectious Diseases, Centers for Disease Control and Prevention, Atlanta, Georgia, USA; 6 Centre for Global Health Research, Kenya Medical Research Institute, Nairobi, Kenya; 7 Institute of Tropical Medicine, Antwerp, Belgium; 8 Respiratory Diseases Branch, Division of Bacterial Diseases, National Center for Immunization and Respiratory Diseases, Centers for Disease Control and Prevention, Atlanta, Georgia, USA; 9 Influenza Program, Centers for Disease Control and Prevention, Nairobi, Kenya

**Keywords:** postmortem interval, molecular diagnosis, pediatric respiratory death, minimally invasive tissue sampling, MITS

## Abstract

**Background:**

We used postmortem minimally invasive tissue sampling (MITS) to assess the effect of time since death on molecular detection of pathogens among respiratory illness–associated deaths.

**Methods:**

Samples were collected from 20 deceased children (aged 1–59 months) hospitalized with respiratory illness from May 2018 through February 2019. Serial lung and/or liver and blood samples were collected using MITS starting soon after death and every 6 hours thereafter for up to 72 hours. Bodies were stored in the mortuary refrigerator for the duration of the study. All specimens were analyzed using customized multipathogen TaqMan® array cards (TACs).

**Results:**

We identified a median of 3 pathogens in each child’s lung tissue (range, 1–8; n = 20), 3 pathogens in each child’s liver tissue (range, 1–4; n = 5), and 2 pathogens in each child’s blood specimen (range, 0–4; n = 5). Pathogens were not consistently detected across all collection time points; there was no association between postmortem interval and the number of pathogens detected (*P* = .43) and no change in TAC cycle threshold value over time for pathogens detected in lung tissue. Human ribonucleoprotein values indicated that specimens collected were suitable for testing throughout the study period.

**Conclusions:**

Results suggest that lung, liver, and blood specimens can be collected using MITS procedures up to 4 days after death in adequately preserved bodies. However, inconsistent pathogen detection in samples needs careful consideration before drawing definitive conclusions on the etiologic causes of death.

Autopsy referrals have declined worldwide despite being the gold standard for determining cause of death [1–3]. In sub-Saharan Africa, most deaths do not undergo postmortem examination [4–7]. Reasons for low utilization of postmortem services in the region include inadequate health facility capacity, costs, and cultural and religious resistance to conventional autopsy practices [5, 8, 9].

Minimally invasive autopsies (MIAs) that involve needle sampling techniques may be a more acceptable means of undertaking postmortem examinations in low- and middle-income countries [8, 10–12]. The MIA approach is gaining popularity in sub-Saharan Africa as a surveillance tool to understand the cause of death, which could then be used to guide prioritization of prevention measures [8, 11, 13, 14]. Nonetheless, we previously observed 42% concordance in pathogen detection from deceased children’s lung tissue collected via minimally invasive tissue sampling (MITS) and conventional autopsy [15]. This emphasizes the need to optimize MITS procedures and understand its utility as a surveillance tool in the current era of increased availability of molecular diagnostics.

Pathogen detection, however, can be affected by the timing of specimen collection [16]. It is recommended that specimen collection for microbiological analyses occur within 24 hours of death to minimize postmortem replication of pathogens and the disintegration of natural barriers that could influence interpretation of laboratory findings due to translocation of pathogens [17, 18]. Unfortunately, this timeline is often unrealistic in resource-limited settings. Limited data exist describing the impact of postmortem sampling intervals on diagnostic performance [16].

In this study, we investigated hospitalized children whose death was associated with respiratory illness to determine the effect of time since death on the detection of pathogens. Here, we describe the frequency and distribution of pathogens detected and discuss the implications of postmortem interval (PMI) for cause of death etiology studies that rely on molecular diagnostics performed on samples collected using MITS techniques.

## METHODS

### Study Site

The study was undertaken at Kenyatta National Hospital, Kenya’s largest teaching and referral hospital, located in the capital city, Nairobi. Pediatric patients admitted to the hospital are either referred from lower-level health facilities within the country or admitted directly through the pediatric outpatient clinic.

From May 2018 to February 2019, we approached the parents/guardians of deceased children aged 1–59 months who were hospitalized with a history of respiratory illness. After written approval from the next of kin, specimens were collected as soon as possible after death, with sequential sample collections occurring at regular intervals of about 6 hours for up to 72 hours. Medical records of the deceased children were retrieved (when available) to obtain clinical information. We aimed to investigate 20 pediatric deaths.

### Sample Collection

The deceased’s body was placed in a body bag and transferred to a dedicated body locker in the mortuary refrigerator. Sample collection commenced as soon as possible after death and was repeated every 6 hours for 9 collections. A final tenth batch of samples was collected 24 hours after the ninth sample.

Bodies were stored in the mortuary refrigerator at 2^o^C–8^o^C from as soon as possible after death and removed briefly during each sample collection. The temperature of the mortuary refrigerator and rectal temperature of the body were recorded before each sample collection. Samples were collected for molecular tests and histopathology assessment; however, histopathologic findings are presented separately.

For molecular analyses, lung samples were collected from all 20 children (case identification P01–P20) at each sample collection encounter using MITS techniques (Supplementary Table 1). Liver tissue and blood specimens (from the heart, subclavian vessels, and femoral vessels) were also collected from a convenience sample of 5 cases (case identification P06–P10).

Before sample collection, surfaces and equipment were cleaned and disinfected with 0.05% and 0.5% sodium hypochlorite solution. During the procedure, the body was exposed but remained inside the body bag, and sample collection sites were cleaned with 70% alcohol solution. Separate, freshly opened, 18-gauge biopsy guns were used to collect lung and liver tissue for molecular testing and histologic examination. Biopsy guns were not reused between sample collections or between children. Eighteen-, 20-, and 22-gauge needles were used to collect blood from the subclavian vessels, femoral vessels, and heart. After completion of sample collection, puncture sites were cleaned with 70% alcohol solution and covered with transparent sterile self-adhesive surgical dressing. The body was then sealed within the body bag and returned to the mortuary refrigerator.

For molecular testing, 1 core each of right and left lung tissue was collected and placed in a single cryovial for each of the 20 children sampled; 2 cores of liver tissue were collected and placed in a single cryovial for each of the 5 children sampled. All cryovials were then placed in a dry shipper with liquid nitrogen. They were later transferred to the laboratory where they were stored at –80^o^C awaiting testing. Blood specimens from 5 children were collected in EDTA-containing vacutainers and placed in a refrigerator for 12–24 hours before being transferred in cool boxes to the laboratory, where they were aliquoted into cryovials and stored at –80^o^C awaiting testing.

For histologic examination, 2 cores of each tissue (right lung, left lung, and liver) were placed in separate sterile containers with 10% buffered formalin solution for 12–18 hours before being transferred to 70% alcohol solution. These samples remained in alcohol until they were transported in 2 consignments to the US Centers for Disease Control and Prevention (CDC) Infectious Diseases Pathology Branch Laboratory (Atlanta, Georgia), where they underwent histologic examination.

### Laboratory Molecular Testing

Lung, liver, and blood samples were tested using TaqMan® array cards (TACs) at the Kenya Medical Research Institute Laboratory. After thawing, 100 µL of blood was used for nucleic acid extraction, while thawed lung and liver tissues were homogenized for 10 minutes in a Sarstedt tube using TissueLyser; 100 µL of the homogenate was then used for nucleic acid extraction. Nucleic acids from blood or tissues were extracted using either MagNA Pure 96 DNA and Viral Nucleic Acid Kit in a MagNA Pure 96 instrument (Roche, Inc) or QIAamp DNA Mini Kit (Qiagen, Inc) following manufacturers’ instructions. TAC testing was carried out as described previously [19] for 42 and 28 pathogens in the respiratory and blood TACs, respectively (Supplementary Table 2). Cards were centrifuged at 1200 revolutions per minute twice for 2 minutes and run in the ViiA-7 polymerase chain reaction (PCR) platform (Applied Biosystems). TAC PCR run results were independently reviewed for quality assurance at the Respiratory Diseases Branch Laboratory, US CDC, and testing was repeated when inconsistencies were detected.

If a pathogen was positive in any 2 testing wells with a cycle threshold (Ct) value <40, it was considered a positive result. Human ribonucleoprotein (RNP), a “housekeeper” gene, was used as a marker for quality of sample handling and processing. Blood specimens were tested for human immunodeficiency virus (HIV) using the Roche Amplicor HIV-1 monitor test kit.

### Data Analysis

Data were analyzed using descriptive statistics. The relationships between time from death and number of lung pathogens detected and TAC Ct value were analyzed using linear regression.

### Ethical Review

Institutional review board (IRB) approval was provided by the Kenya Medical Research Institute and Kenyatta National Hospital–University of Nairobi. The study was exempt from US CDC IRB review as it did not involve live human subjects.

## RESULTS

### Case Enrollment and Clinical Information

We approached 33 families to achieve our target sample size of 20 (61% acceptance rate). Fifteen of the children included in the study were male. The median age of children was 8 months (range, 1–53). For 18 children where clinical information was available, the median time from admission to death was 1 day (range, 0–67). Six cases (33%) had received blood transfusions during admission, and only 1 patient was tested for HIV before death and was found to be negative.

The most common comorbidities documented in medical files were presence of malnutrition (n = 3, 17%), chronic respiratory disease (n = 3, 17%), and chronic neurological disorders including convulsive disorders and cerebral palsy (n = 2, 11%). The most common clinical diagnoses were pneumonia (n = 14, 78%), followed by meningitis/encephalitis (n = 5, 28%) and gastroenteritis (n = 4, 22%; Table 1).

**Table 1.** Characteristics of Children Enrolled in Postmortem Study Conducted Following Respiratory Illness–Associated Death

Characteristic	Value
Median age (range), months	8 (1–53)
Gender, n (%)	
Male	15 (75)
Female	5 (25)
Median weight (range), kg	6.6 (3.6–10.1)
Weight categories,^a^ n (%)	
Normal weight	10 (50)
Underweight	5 (25)
Severely underweight	5 (25)
Median duration from admission to death (range), days	1 (0–67)
Comorbidities, n (%)	
None documented	12 (67)
Protein energy malnutrition	3 (17)
Chronic respiratory disease (eg, pulmonary arterial hypertension^b^)	3 (17)
Chronic neurological or neuromuscular disorders (eg, convulsive disorder, cerebral palsy)	2 (11)
Newly diagnosed tuberculosis	1 (6)
Congenital heart disease (in this case mesocardia)	1 (6)
Rickets	1 (6)
Clinical diagnoses at time of death,^c^ n (%)	
Pneumonia/bronchopneumonia	14 (78)
Meningitis/encephalitis	5 (28)
Gastroenteritis/diarrhea	4 (22)
Sepsis	3 (17)
Malnutrition	3 (17)
Shock	3 (17)
Dehydration	2 (11)
Bronchitis	2 (11)
Congenital heart disease	2 (11)
Acute respiratory distress syndrome	1 (6)
Tuberculosis	1 (6)
Anemia	1 (6)
Acute kidney injury	1 (6)
Metabolic acidosis	1 (6)
Laryngotracheobronchitis	1 (6)
Cerebral palsy	1 (6)
Down’s syndrome	1 (6)
Severe pulmonary artery hypertension	1 (6)

^a^ Weight-for-age categories based on the local growth monitoring chart provided by the Ministry of Health, Kenya.

^b^ A description of the specific chronic respiratory disease was not available for 2 of 3 children.

^c^ Multiple responses permitted, and total denominator reflected 18 children whose medical records were accessible to the study staff.

### PMI and Temperature Monitoring

**Figure 1.**
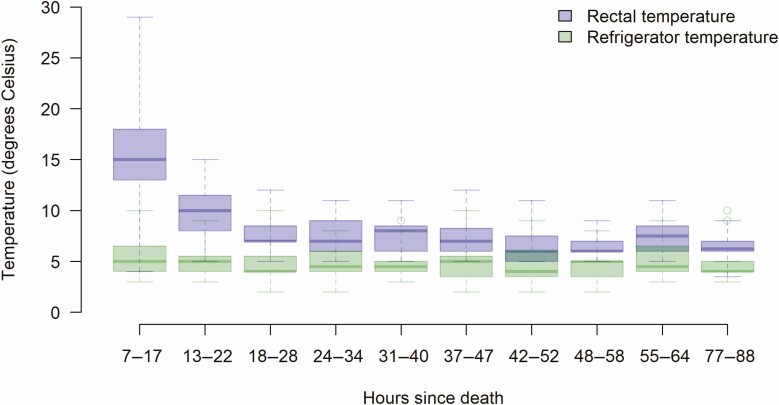
Box plot of refrigerator and rectal temperatures at each batch of sample collection for 20 children.

The median PMI to first sample collection was 12.25 hours (range, 7.25–17). The median PMI to last sample collection was 83.33 hours (range, 77.25–88.33). The median refrigerator temperature recorded before each sample collection was 5^o^C and ranged from 2^o^C to 10^o^C. The median rectal body temperature was 15^o^C at the first sample collection and dropped to 7^o^C by the third sample collection (12 hours after first sample collection; Figure 1).

### Blood Collection

Blood collection was considered successful if at least 0.5 mL of blood was collected. Collection of cardiac blood was successful in 68% (34 of 50) of attempts. Blood collection was successful in 56% (28 of 50) of attempts from subclavian vessels and 28% (14 of 50) of attempts from femoral vessels. Notably, successful blood collections could occur after previous unsuccessful attempts on the same child (Supplementary Figure 1). Among these cases, blood collection from the heart was successful up to 83.5 hours after death, while blood collection from the subclavian vessels and femoral vessels was successful up to 62.75 and 33.5 hours after death, respectively.

### Molecular Test Results

#### Frequency and Distribution of Pathogen Detection

**Figure 2.**
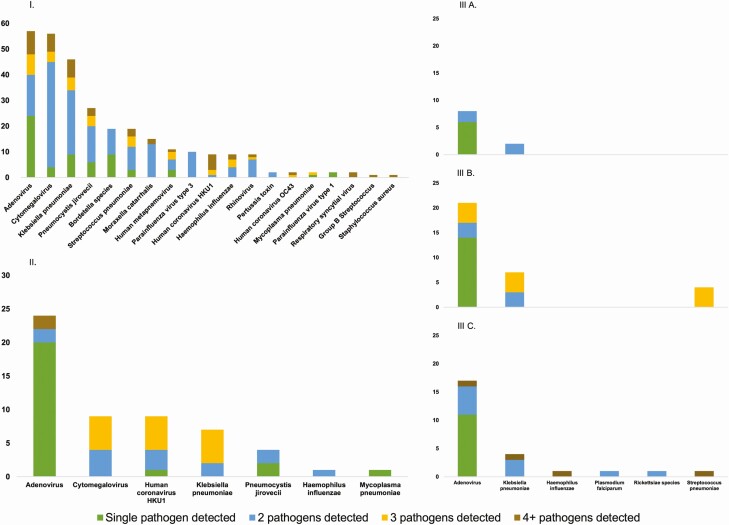
Frequency of pathogens detected. *(I)* Nineteen pathogens detected in lung specimens from 20 children. *(II)* Seven pathogens detected in liver specimens from 5 children. *(III)* Six pathogens detected in femoral blood specimens *(A)*, cardiac blood specimens *(B)*, and subclavian blood specimens *(C)* from 5 children.

At least 1 pathogen was detected in the lung tissue of all 20 children and liver tissue of all 5 children tested. However, pathogens were detected in blood samples of only 3 of the 5 children tested. Notably, if pathogens were detected in a lung specimen, it was common to detect more than 1 pathogen (Figure 2). In lung specimens where pathogens were detected, 62% of these lung specimens had multiple pathogens detected with a median of 2 pathogens detected per specimen. Conversely, multiple pathogen detection was less common in liver (24%) and blood specimens (25%–35%) where usually 1 pathogen was detected if a pathogen was detected in either of these specimens (Figure 2).

Nineteen pathogens were detected among 82% (162 of 198) of lung specimens tested (Figure 2), and no pathogens were detected in 18% (36 of 198). Among the 5 children where liver and lung specimens were collected for molecular testing, 11 pathogens were detected in 94% (47 of 50) of lung specimens and 7 pathogens were detected among 72% (36 of 50) of liver specimens (Figure 2). Notably, if a pathogen was detected in the liver, 91% of the time it was also identified in the lung, although not always from the same specimen collection time point. One exception was *Mycoplasma pneumoniae* detected in the first batch of liver specimens collected from case P06 but not in any lung specimens collected from the same child.

Six pathogens were detected in 63% (17 of 27) of subclavian blood specimens tested, while 3 pathogens were detected in 62% (21 of 34) of cardiac blood specimens and 2 pathogens were detected in 62% (8 of 13) of femoral blood specimens (Figure 2).

Overall, when considering each specimen type separately, we identified a median of 3 pathogens in each child’s lung tissue (range, 1–8; n = 20), 3 pathogens in each child’s liver tissue (range, 1–4; n = 5), and 2 pathogens in each child’s blood specimen (range, 0–4; n = 5). For the 6 children who received blood transfusions during admission, we identified a median of 4 pathogens in each child’s lung tissue (range, 2–5; n = 6) and 1 pathogen in each child’s liver (range, 1; n = 2) and blood specimens (range, 0–2; n = 2).

For the 5 children where blood, liver, and lung specimens were collected, a median of 5 (range, 4–8) unique pathogens were detected per child, and 1 was positive for HIV.

#### Consistency of Pathogen Detection and the Effect of Time

**Figure 3.**
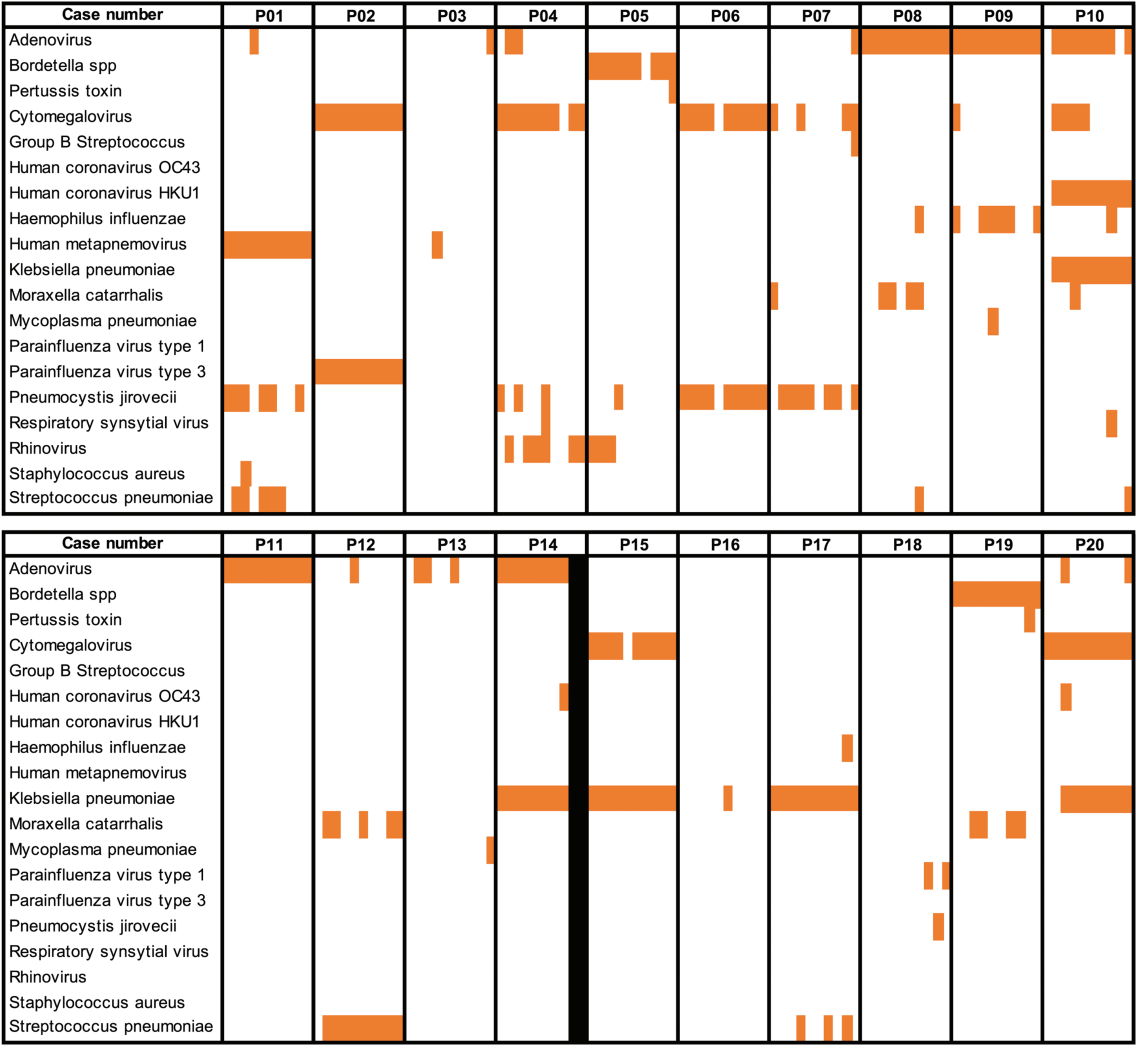
Schematic showing frequency of pathogen detection for each child across all batches of lung specimens tested. Orange cells indicate TaqMan array card (TAC)–positive specimens. White cells indicate TAC-negative specimens. Black cells indicate specimens from patient P14 (batches 9 and 10) that were not tested.

Pathogens were not consistently detected across all time points. Figure 3 illustrates the consistency in detection of pathogens in lung specimens per child over the course of sequential sampling. Adenovirus, for instance, although detected in the lung tissue of 12 children, was not consistently detected throughout sample collection batches. In a few cases, pathogens were consistently detected across all specimens tested from the same child (eg, human metapneumovirus in P01, cytomegalovirus in P02, and adenovirus in P08). Generally, if a pathogen was detected in a child, it was only detected in half of the lung and liver specimens tested from that child. If a pathogen was detected in femoral, cardiac, or subclavian blood, it was detected in 75%, 67%, and 50%, respectively, of the blood specimens tested from the child.

Using linear regression, we found no association between PMI and the number of pathogens overall (*P* = .43) or between PMI and the number of bacterial pathogens (*P* = .33) detected in lung tissue. We observed no change in the TAC Ct value over time for pathogens detected in lung tissue (Supplementary Figure 2). There was no significant change in human RNP Ct over time for each of the sample types tested; values ranged from 17 to 30, indicating stable quality of the samples (Supplementary Figure 3).

The Supplementary Material depicts the consistency of pathogen detection over time across different types of specimens (Supplementary Figure 4). Concordance of molecular test results between specimen types was variable, with organisms such as human coronavirus HKU1, *Klebsiella pneumoniae*, and adenovirus having good levels of concordance between different types of specimens, while other, less frequently identified organisms usually had lower levels of concordance (Supplementary Table 3).

## Discussion

In this study, we are the first to examine the impact of PMI on pathogen detection through sequentially collected postmortem lung, liver, and blood specimens from the same child using MITS. In our study, there was no change in the number of pathogens detected or significant change in pathogen concentration (measured through Ct values) over time for up to 96 hours after death when adequate body storage conditions and aseptic specimen collection techniques were followed. Amplifiable human RNP with Ct values between 17 and 30 was obtained consistently during sample collection, indicating that specimens were likely to be suitable for testing up to 4 days after death. However, pathogens were not consistently detected in all sequential specimens tested from the same child. This inconsistency in detection could represent localized pathogenesis or amplification of microbial particles representing past infections or colonization, not necessarily associated with the current pathological process. Multiple and inconsistent pathogen detection can make interpretation of etiological studies challenging, especially if the extent of lung pathology was not observed in conventional autopsy or if histopathology readings are not available to aid interpretation.

We followed procedures to avoid cross-contamination and successfully maintained the ideal temperature conditions to allow for the collection of quality samples up to 4 days after death, avoiding commonly described bacterial translocation and overgrowth [20]. A study conducted in Mozambique to assess the impact of PMI on the diagnostic performance of MIAs comparing autopsies conducted before and after 24 hours following death concluded that after 24 hours, bacterial overgrowth could mislead conclusions of the cause of death [16]. However, unlike our study, they were not able to control for differences in individual patient’s characteristics, as well as body storage conditions that could have explained the increase in the number of bacteria detected beyond a PMI of 24 hours in their study.

Our findings support MITS protocols that use multiple specimens from parenchymal organs [21], thus increasing the likelihood of detecting key pathologic changes and associated etiologies, especially if the pathologic process is localized. However, as we showed in our results, 1 batch of specimens collected during a particular time point may fail to detect relevant pathogens. For example, in our study, if a pathogen was detected in a child, it was only detected in half of the lung and liver specimens tested from that child even though the number of pathogens detected did not vary with time since death. Moreover, for each type of specimen examined, multiple pathogens were identified. These findings are important when considering an MITS technique as the sole approach to investigate etiology associated with cause of death because several pathogens are often detected and accurate correlation with cause of illness or death requires one to consider clinical information and other laboratory findings inclusive of molecular tests, culture, and histopathology results [11, 15, 22, 23].

More than 1 pathogen can contribute to death due to infectious causes in children [11]. In a case-control study comparing pathogens detected in nonfatal and fatal pediatric respiratory disease cases that were matched to healthy asymptomatic controls, the authors identified multiple pathogens in all 3 groups, making interpretation of results challenging [24]. Although nasal and oropharyngeal swabs were collected while children were alive, this and other studies highlight the complexity of attributing the etiology of respiratory illness [11, 22, 23]. Very sensitive molecular techniques can detect microorganisms or microbial RNA/DNA in clinical specimens; however, this does not necessarily imply a causal relationship. Adenovirus is an interesting example as the virus is associated with various clinical manifestations and is detected in multiple tissues (eg, conjunctival, respiratory, gastrointestinal, urinary, brain, and cardiac tissues). Nonetheless, researchers have indicated that adenovirus may persist at low levels in humans where molecular detection may be coincidental rather than causal, especially in persons with weakened immune systems, as could be the case in our study population [25]. The concomitant identification of adenoviruses in lung, liver, and blood specimens might corroborate a causal association. However, additional studies to improve our understanding of molecular diagnostics in detecting pathogens with the ability to be dormant in human tissues are warranted.

In our study, we demonstrated that blood from the heart could be collected up to 3.5 days after death and was more often successfully collected than blood from other sites. Generally, in forensic autopsy practice, peripheral blood collection sites are preferred to cardiac blood when conducting biochemical and toxicology analyses [17, 26, 27]. However, we have previously observed that for infectious disease diagnostics, cardiac blood is unlikely to provide inaccurate assessment and may be used when peripheral blood collection is unsuccessful [28, 29].

Our study had some limitations. Our findings cannot be extrapolated to the effect of PMI on bodies maintained at room temperature. This is especially relevant in settings where deaths occur in the community and bodies are not refrigerated soon after death that could lead to bacterial translocation and overgrowth [20, 30]. Also, we chose to perform serial sample collections from each child to allow only time since death to affect pathogen detection, in effect using each child as its own control. However, there were inconsistencies in pathogen detection across sample collection batches. Perhaps, a larger sample size could facilitate exploring some of these inconsistencies, especially if blood and liver specimens had been collected for all participants.

In conclusion, our data suggest that MITS specimens collected for multipathogen molecular testing can provide reliable results for up to 96 hours after death if adequate body storage conditions and aseptic specimen collection techniques are followed. Nonetheless, our findings raise important concerns as pathogens were detected inconsistently across sample collection batches. This demonstrates the need for careful interpretation of molecular results that may benefit from concomitant histopathological investigation when attempting to determine the etiology of infectious causes of death.

## Supplementary Data

Supplementary materials are available at *Clinical Infectious Diseases* online. Consisting of data provided by the authors to benefit the reader, the posted materials are not copyedited and are the sole responsibility of the authors, so questions or comments should be addressed to the corresponding author.

ciab810_suppl_Supplementary_Materials

## Notes

***Author contributions.*** S. S. C., M. A. W., and S. Z. conceptualized the study and, with R. M. and N. O., developed the protocol. S. S. C. supervised the implementation of the study. J. D., E. W., J. M., P. M., M. B., W. O., and W. B. conducted sample and data collection. C. O., L. M., M. O., V. O., S. L., E. H., M. D., and J. W. conducted laboratory testing. J. D. conducted data analysis. J. D., E. W., C. O., J. S., L. M., M. O., S. L., E. H., N. O., J. R., M. A. W., M. D., J. W., R. M., S. Z., and S. S. C. interpreted the data. J. D. and S. S. C. developed the first draft of the manuscript. All authors reviewed the manuscript.

***Acknowledgments.*** The authors thank the following individuals for their technical support: Gideon Emukule (Centers for Disease Control and Prevention [CDC], Kenya), M. Kariuki Njenga (Washington State University), Bryan Nyawanda (Kenya Medical Research Institute), Kathy Banke (Bill & Melinda Gates Foundation, USA), Scott Dowell (Bill & Melinda Gates Foundation, USA), Cynthia Whitney (Emory University), Dianna Blau (US CDC), and Jennifer Verani (US CDC). In addition, the authors thank the Kenyatta National Hospital for the support provided during the study and the families of the deceased children for agreeing to their child’s enrollment in the study. All data analyzed during this study can be made available by the corresponding author upon reasonable request.

***Supplement sponsorship.*** This supplement is sponsored by MITS Surveillance Alliance Secretariat, led by RTI International, with funding from the Bill and Melinda Gates Foundation.

***Financial support.*** This work was supported, in whole, by the Bill & Melinda Gates Foundation (OPP1168515). Under the grant conditions of the foundation, a Creative Commons Attribution 4.0 Generic License has already been assigned to the Author Accepted Manuscript version that might arise from this submission.

***Potential conflict of interest.*** S. S. C. has since left the CDC and is currently an employee of Sanofi Pasteur. All remaining authors: No reported conflicts of interest. All authors have submitted the ICMJE Form for Disclosure of Potential Conflicts of Interest. Conflicts that the editors consider relevant to the content of the manuscript have been disclosed.
