# Supplementary material for: Effect of Time Since Death on Multipathogen Molecular Test Results of Postmortem Specimens Collected Using Minimally Invasive Tissue Sampling Techniques
Source: Clin Infect Dis. 2021 Dec 15;73(Suppl 5):S360–7. doi: 10.1093/cid/ciab810 (PMC8672767; doi:10.1093/cid/ciab810)
Supplement: ciab810_suppl_Supplementary_Materials [file ciab810_suppl_supplementary_materials.docx]

**Supplementary material**

**Effect of time since death on multi-pathogen molecular test results of postmortem specimens collected using minimally invasive tissue sampling techniques**

Jeanette Dawa, Edwin Walong, Clayton Onyango, John Mathaiya, Peter Muturi, Milka Bunei, Washington Ochieng, Walter Barake, Josilene N. Seixas, Lillian Mayieka, Melvin Ochieng, Victor Omballa, Shirley Lidechi, Elizabeth Hunsperger, Nancy Otieno, Jana M. Ritter, Marc-Alain Widdowson, Maureen H. Diaz, Jonas M. Winchell, Roosecelis Martines, Sherif R. Zaki, Sandra S. Chaves

**Table S1: Procedure for sample collection at each site**

| **Sample** | **Procedure** |
| --- | --- |
| **Skin swab** | At the start of sample collection before cleaning of the body, a sterile cotton swab was rubbed along the subject’s skin over the upper and lower body and the tip placed in a cryovial. |
| **Subclavian blood** | The needle was inserted superior to the clavicle and 1 cm lateral to the sternocleidomastoid muscle while aiming inferiorly (towards the clavicle) at an angle of 30 degrees. The needle was then advanced in a plane almost parallel to the skin approximately 2-3 cm. The angle of penetration of the needle was changed until blood was freely aspirated into the syringe. In case no blood was obtained with this supraclavicular approach, we used an infra-clavicular puncture aiming to reach the subclavian vein behind the clavicle. |
| **Cardiac blood** | The needle was inserted at the left parasternal fifth intercostal space at the midpoint of the clavicle. The needle was inserted 5-6 cm. In case no blood was obtained, we used the 4^th^ and 3^rd^ left parasternal intercostal spaces. |
| **Femoral blood** | The needle was inserted at the midpoint of the right inguinal line and moved up and down and re-angled until blood was aspirated. If unsuccessful, an attempt was made to obtain blood at the midpoint of the left inguinal line. |
| **Right and left lung specimens** | The biopsy gun needle was inserted into the right/left mid-clavicular line, upper thoracic region (around 2^nd^ intercostal space). The needle was penetrated as much as possible in a quick movement. After reaching the limit, the biopsy gun was retracted 2-3 cm depending on the size of the chest capacity. The biopsy gun was then engaged, and the needle withdrawn. The tissue collected was examined and expected to be 20 by 1 mm in size, of a pink/reddish color and soft consistency. If lung tissue was not obtained the procedure was repeated, and the needle angled in a different direction. |
| **Liver specimen** | The biopsy gun needle was inserted in the right mid-axillary line, in any one of the last three intercostal spaces. The needle was oriented 30 degrees in a cranial direction and inserted 2-5 cm depending on the age of the patient. The biopsy gun was then engaged, and the needle withdrawn. The specimen was confirmed to be about 20 by 1 mm in size and brownish in color. If not, the procedure was repeated, and the needle angled slightly towards the expected position of the liver on entry. |
| **Body bag swab** | At the end of sample collection, after sample collection sites had been sealed with self-adhesive surgical dressing, the inside of the body bag was swabbed with a sterile cotton swab and the tip placed in a cryovial. |

**Table S2: List of pathogens tested in blood and respiratory TAC**

| **Pathogen** | **Blood TAC card** | **Lung TAC card** |
| --- | --- | --- |
| Acinetobacter baumanii |  | x |
| Adenovirus | x | x |
| B. parapertussis, B. bronchiseptica |  | x |
| Bordetella spp (B. pertussis or B. holmseii) |  | x |
| Burkholderia pseudomallei |  | x |
| Chikungunya virus | x |  |
| Chlamydia pneumoniae |  | x |
| Chlamydia trachomatis |  | x |
| Corynebacterium diphtheriae |  | x |
| Corynebacterium pseudotuberculosis |  | x |
| Corynebacterium spp. (tox gene) |  | x |
| Cryptococcus neoformans, Cryptococcus gattii | x |  |
| Cytomegalovirus |  | x |
| Dengue virus | x |  |
| Enterovirus | x | x |
| Escharichia coli/Shigella | x |  |
| Group A Streptococcus | x | x |
| Group B Streptococcus | x | x |
| Haemophilus influenza | x | x |
| Haemophilus influenzae type B | x |  |
| Human coronavirus 229E |  | x |
| Human coronavirus NL63 |  | x |
| Human coronavirus OC43 |  | x |
| Human coronavirus HKU1 |  | x |
| Human metapneumovirus |  | x |
| Influenza A |  | x |
| Influenza B |  | x |
| Klebsiella pneumoniae | x | x |
| Listeria monocytogenes | x |  |
| Measles |  | x |
| MERS coronavirus (N2 gene) |  | x |
| MERS coronavirus (upE gene) |  | x |
| Moraxella catarrhalis |  | x |
| Mycobacterium pneumoniae |  | x |
| Mycoplasma pneumoniae |  | x |
| Neisseria meningitides | x |  |
| Orientia tsutsugamushi | x |  |
| Parainfluenza virus type 1 |  | x |
| Parainfluenza virus type 2 |  | x |
| Parainfluenza virus type 3 |  | x |
| Parainfluenza virus type 4 |  | x |
| Parenchovirus | x |  |
| Pertussis toxin - B. parapertussis or B. pertussis |  | x |
| Plasmodium falciparum | x |  |
| Plasmodium vivax | x |  |
| Pneumocystis jirovecii |  | x |
| Pseudomonas aeruginosa | x | x |
| Respiratory syncytial virus |  | x |
| Rhinovirus |  | x |
| Rickettsiae species | x |  |
| Rubella virus | x | x |
| Salmonella enterica Typhi | x |  |
| Salmonella paratyphi A | x |  |
| Salmonella species | x |  |
| Staphylococcus aureus | x | x |
| Streptococcus pneumoniae | x | x |
| Toxoplasma gondii | x |  |
| Treponema pallidum | x |  |
| Varicella zoster virus |  | x |
| Zika virus | x |  |


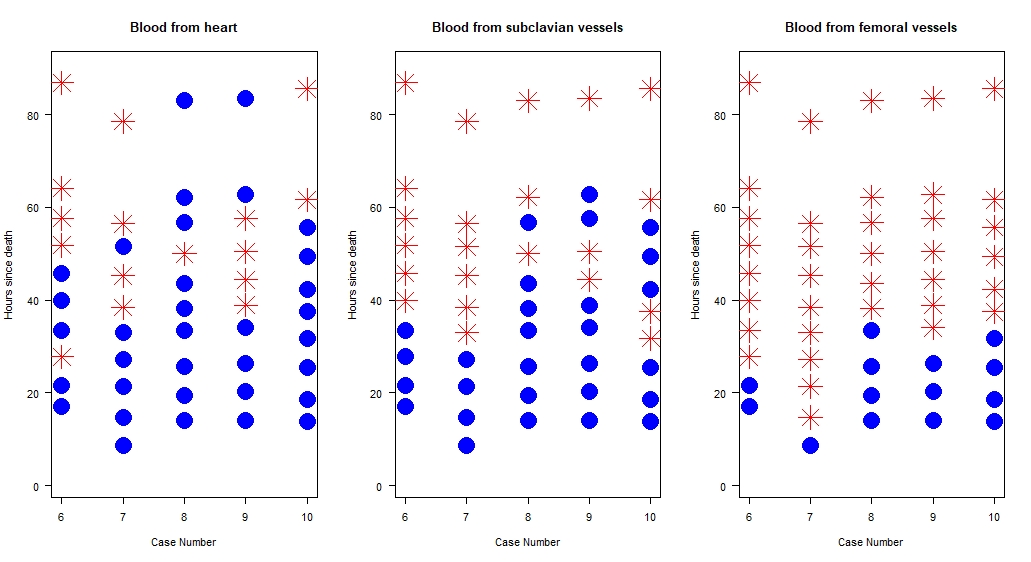


**Figure S1: Frequency of blood collection for 5 subjects. Blue dots refer to batch collections where sample collection was successful, and the red stars indicate blood collection was unsuccessful**


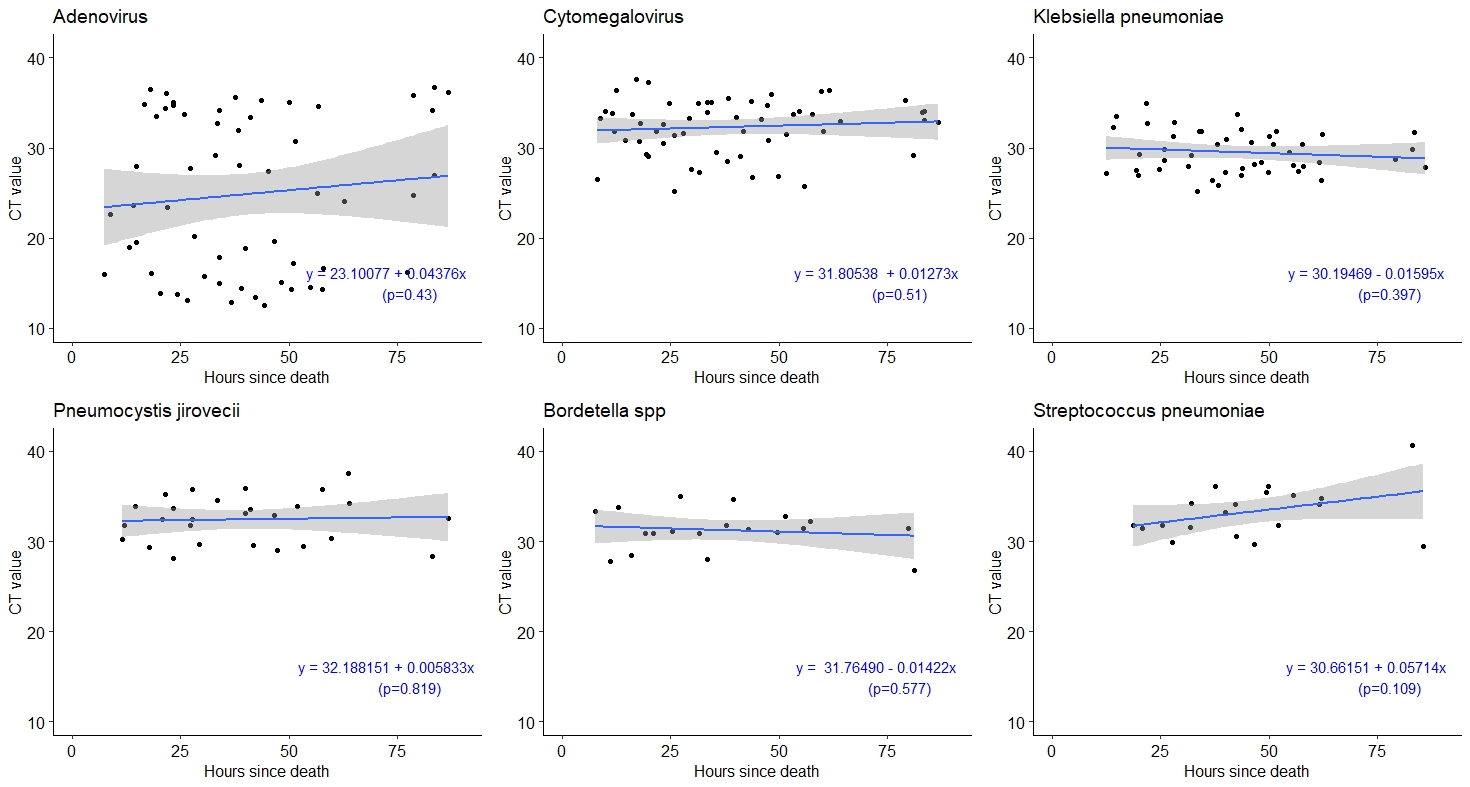


**Figure S2: Cycle threshold TAC test values of the most frequently detected pathogens over time.**


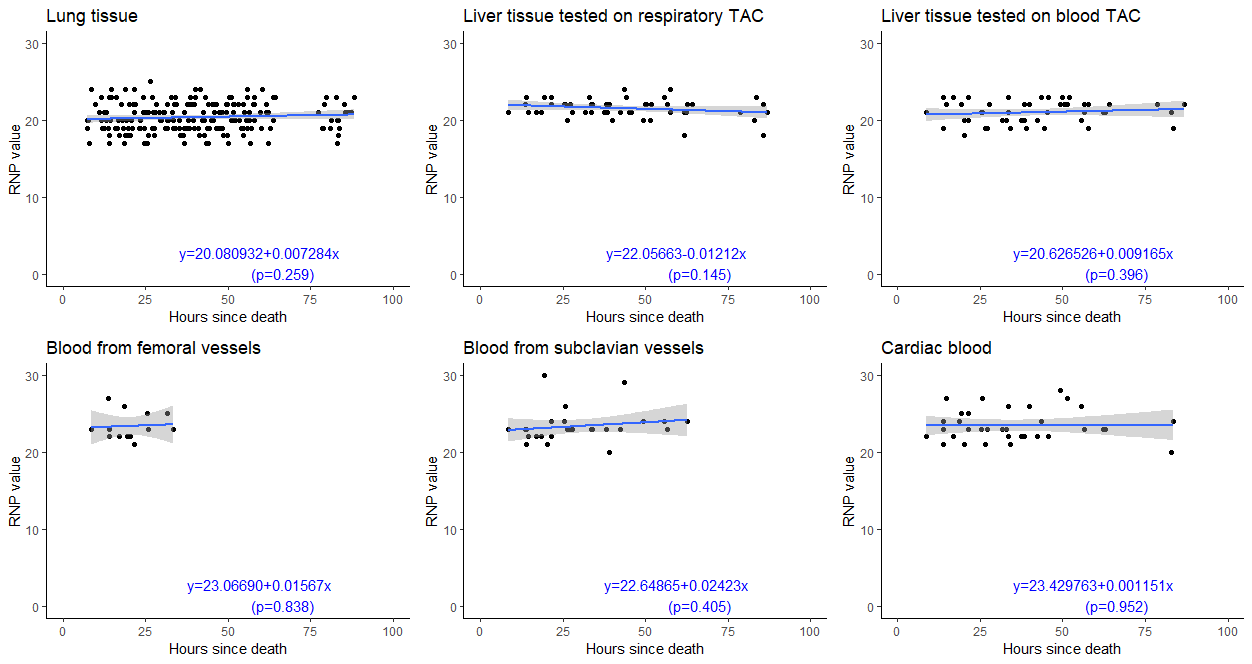


**Figure S3: Human ribonucleoprotein cycle threshold values for tissue specimen undergoing TAC testing.**


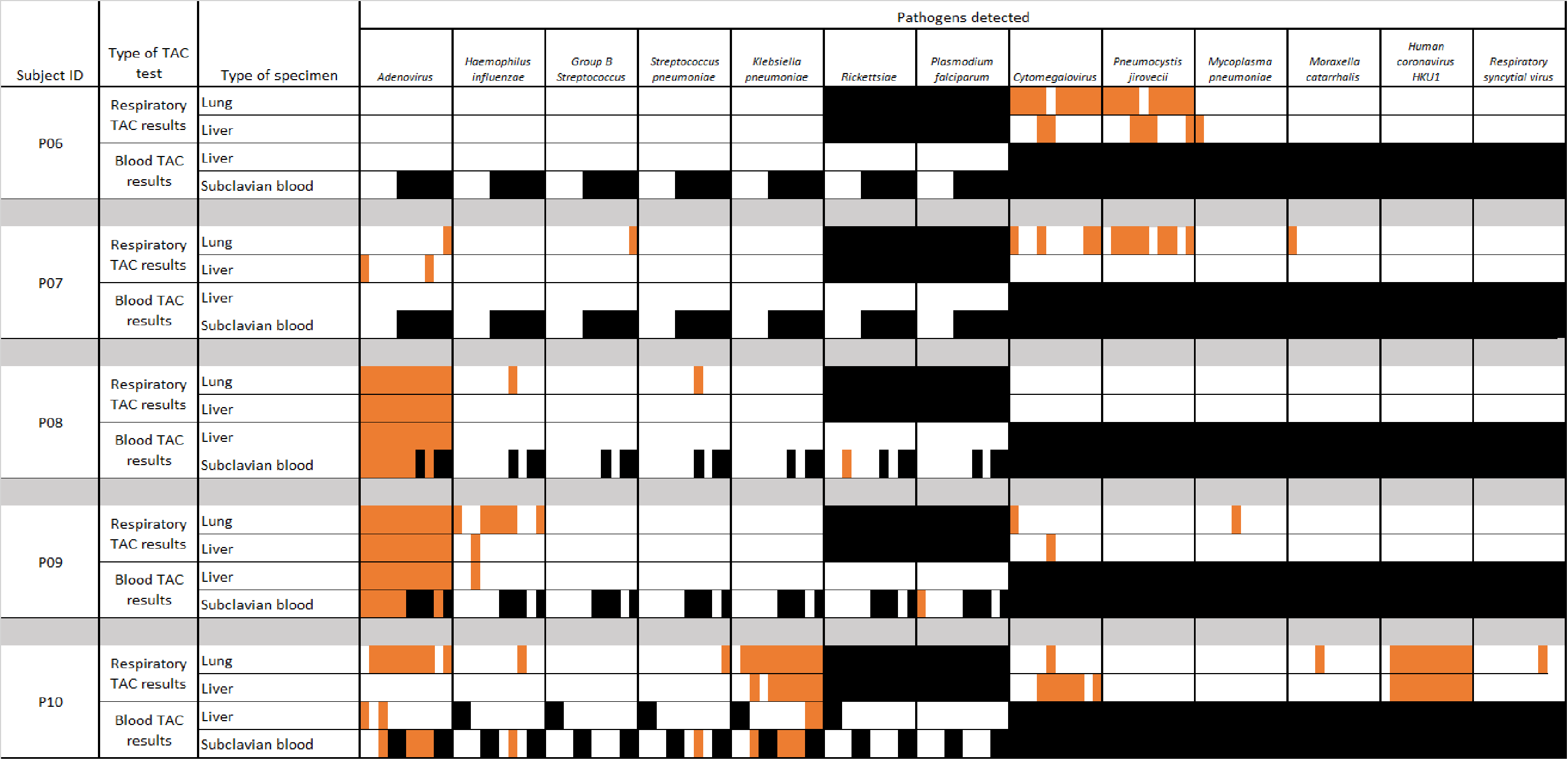


**Figure S4:** **Schematic showing frequency of pathogen detection for each subject (P06-P10) across all batches of lung, liver and subclavian blood tested. Orange cells indicate TAC positive specimens. White cells indicate TAC negative specimens. Black cells indicate specimens that were not tested either because the sample was not collected, or the pathogen referred to was not part of the respiratory or blood TAC test.**

**Table S3: Concordance of molecular test results between combinations of lung, liver and blood specimen**

| **Pathogen** | **Agreement** | | |  | **Concordance** | | |
| --- | --- | --- | --- | --- | --- | --- | --- |
|  | **All specimen positive** | **All specimen negative** | **Overall percent agreement** |  | **Fleiss Kappa value** | **p value** | **Interpretation** |
| **Concordance of molecular test results between 50 pairs of lung and liver specimen tested with respiratory TAC** | | | | | | | |
| Human coronavirus HKU1 | 9 | 41 | 100% |  | 1 | <0.0001 | Perfect agreement |
| *Klebsiella pneumoniae* | 7 | 41 | 96% |  | 0.851 | <0.0001 | Almost perfect agreement |
| Adenovirus | 20 | 19 | 78% |  | 0.56 | <0.0001 | Moderate agreement |
| *Pneumocystis jirovecii* | 3 | 33 | 72% |  | 0.125 | 0.377 | Slight agreement |
| *Haemophilus influenzae* | 0 | 41 | 82% |  | -0.0989 | 0.484 | Poor agreement |
| Group B Streptococcus | 0 | 43 | 86% |  | -0.0654 | 0.606 | Poor agreement |
| Cytomegalovirus | 3 | 26 | 58% |  | -0.0654 | 0.644 | Poor agreement |
| *Moraxella catarrhalis* | 0 | 44 | 88% |  | -0.0638 | 0.652 | Poor agreement |
| *Streptococcus.pneumoniae* | 0 | 48 | 96% |  | -0.0204 | 0.885 | Poor agreement |
| *Mycoplasma pneumoniae* | 0 | 48 | 96% |  | -0.0204 | 0.885 | Poor agreement |
| Respiratory Syncytial Virus | 0 | 49 | 98% |  | -0.0101 | 0.943 | Poor agreement |
|  |  |  |  |  |  |  |  |
| **Concordance of molecular tests results between 25 pairs of liver specimen and subclavian blood tested with blood TAC** | | | | | | | |
| Adenovirus | 13 | 8 | 84% |  | 0.667 | 0.0009 | Substantial agreement |
| *Klebsiella pneumoniae* | 0 | 21 | 84% |  | -0.087 | 0.664 | Poor agreement |
| Rickettsiae | 0 | 24 | 96% |  | -0.0204 | 0.919 | Poor agreement |
| *Plasmodium falciparum* | 0 | 24 | 96% |  | -0.0204 | 0.919 | Poor agreement |
| *Haemophilus influenzae* | 0 | 23 | 92% |  | -0.0417 | 0.835 | Poor agreement |
| *Streptococcus.pneumoniae* | 0 | 24 | 96% |  | -0.0204 | 0.919 | Poor agreement |
|  |  |  |  |  |  |  |  |
| **Concordance of molecular test results between 12 combinations of cardiac blood, subclavian blood and femoral blood tested with blood TAC** | | | | | | | |
| Adenovirus | 7 | 5 | 100% |  | 1 | <0.0001 | Perfect agreement |
| *Klebsiella pneumoniae* | 1 | 11 | 100% |  | 1 | <0.0001 | Perfect agreement |
| Rickettsiae | 0 | 11 | 92% |  | -0.0286 | 0.864 | Poor agreement |
| *Plasmodium falciparum* | 0 | 11 | 92% |  | -0.0286 | 0.864 | Poor agreement |
|  |  |  |  |  |  |  |  |
| **Concordance of molecular test results between 27 pairs of lung tissue and subclavian blood for pathogens common to respiratory and blood TAC** | | | | | | | |
| Adenovirus | 17 | 9 | 96% |  | 0.919 | <0.0001 | Almost perfect agreement |
| *Klebsiella pneumoniae* | 4 | 22 | 96% |  | 0.867 | <0.0001 | Almost perfect agreement |
| *Haemophilus influenzae* | 0 | 22 | 82% |  | -0.102 | 0.596 | Poor agreement |
| *Streptococcus.pneumoniae* | 0 | 26 | 96% |  | -0.0189 | 0.922 | Poor agreement |
